# P-2254. Incidence, Clinical Characteristics and Outcomes of Heart Transplant Patients with Mucormycosis Infection: A Systematic Review and Meta-Analysis

**DOI:** 10.1093/ofid/ofae631.2407

**Published:** 2025-01-29

**Authors:** Austin Auyeung, Kristine-Lou Gargaritano, Frank Doyle, Sanil Thomas

**Affiliations:** University of Central Florida College of Medicine/HCA Florida North Florida Hospital, Gainesville, Florida; Mount Sinai Morningside-West Hospitals, Icahn School of Medicine at Mount Sinai, New York, New York; Royal College of Surgeons in Ireland, Dublin, Dublin, Ireland; HCA Florida North Florida Hospital, Gainesville, Florida

## Abstract

**Background:**

Mucormycosis confers high morbidity and mortality in the immunocompromised population due to its rapid angio-invasive nature. Of note, this group of people includes those who have undergone solid organ or stem cell transplants, many of whom are placed on chronic immunosuppressive regimens. Mucormycosis incidence and clinical outcomes have been studied in bone marrow, lung, liver and kidney transplant recipients but not in heart transplant patients. Thus, our aim with this systematic review and meta-analysis is to characterize the incidence as well as clinical outcomes of Mucor infections in heart transplant recipients.

**Figure 1.**
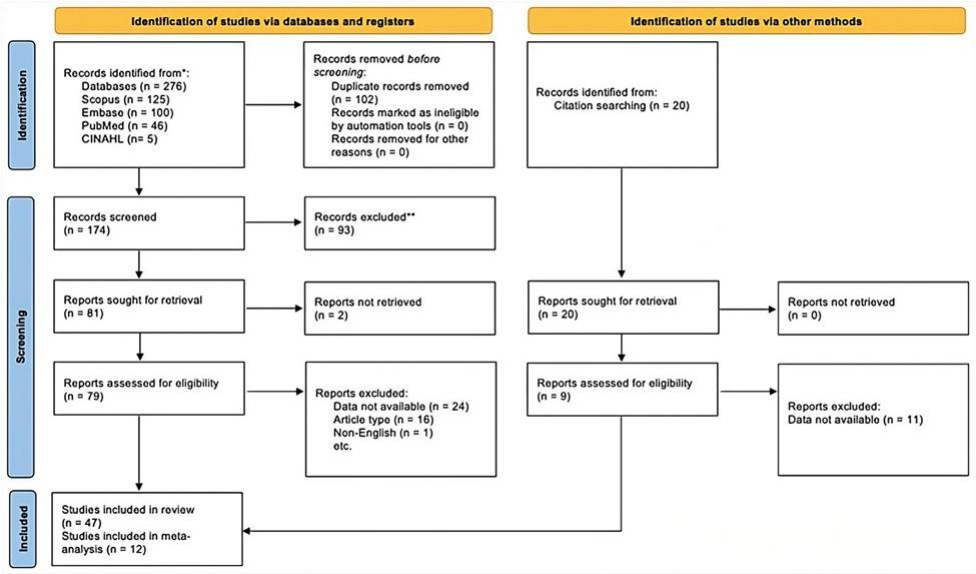
PRISMA Flowchart

**Methods:**

This study was registered with PROSPERO. Electronic databases Google Scholar, PubMed, Embase, Scopus and CINAHL were searched from inception up to 1 February 2024 for peer-reviewed articles of heart transplant patients who contracted mucormycosis. The primary and secondary outcome measures were incidence of mucormycosis infection and times to onset of infection and mortality, respectively. For statistical analysis, overall meta-analytic incidence was estimated using the user-written *metaprop_one[FD1]* [m2] command in Stata 15.1.

**Table 1.**
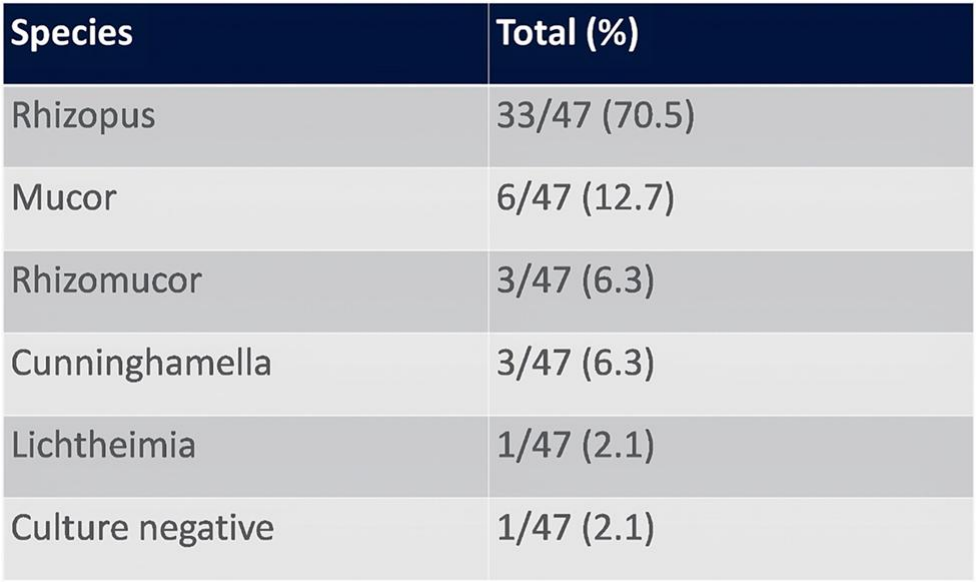
Species Identified in Infection

**Results:**

Our search identified 296 studies, 88 of which underwent full-text review and 47 met inclusion criteria with 3916 total cases (Figure 1). Rhizopus was the most common pathogen identified causing mucormycosis infection (Figure 2). Pooled incidence of mucormycosis infection in heart transplant patients is 0.6% (95% CI 0 - 2%) across 12 studies (Figure 3). The majority of patients were male, 39/41 (95%) (Figure 4). Mucormycosis infection most commonly occurred during first 30 days post-transplant and decreased with time. Logistic regression analysis did not show significant association between time of infection post-transplant and mortality: Odds Ratio = 0.89 (95% CI 0.55-1.42, p = 0.616).

**Figure 2.**
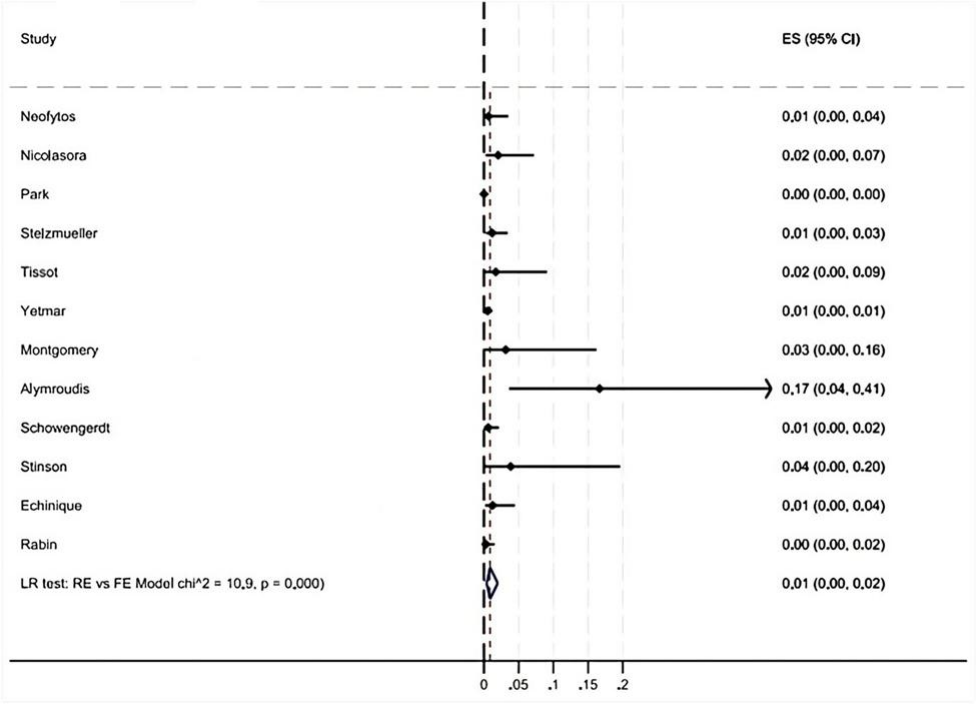
Results of Meta-Analysis for Incidence of Mucormycosis Infection in Heart Transplant Recipients

**Conclusion:**

Overall incidence of mucormycosis in heart transplant patients is 0.6%. Patients were more likely to contract the infection early (within 30 days) in the post-transplant period but there was no significant difference in mortality over time. The majority of affected patients were male and further studies are needed to determine if males are at higher risk of infection.

**Table 2.**
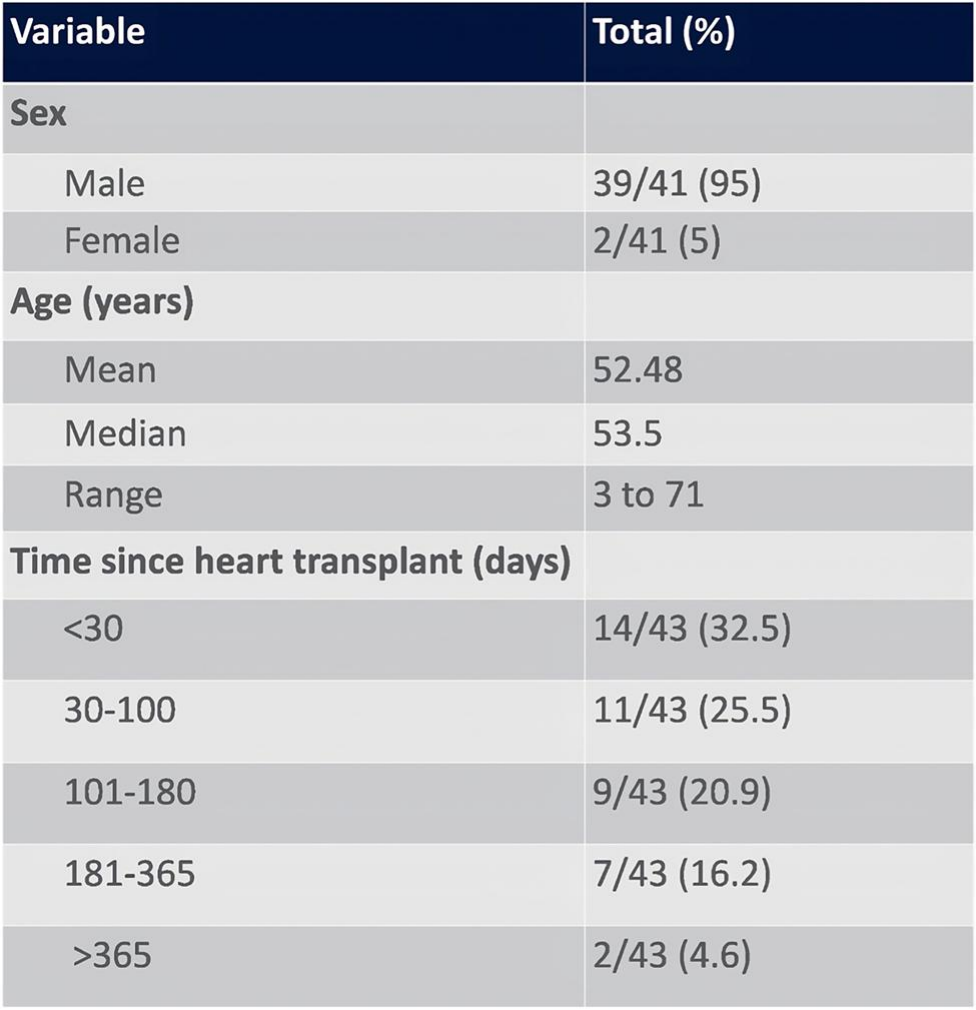
Demographics and Time of Infection Onset

**Disclosures:**

All Authors: No reported disclosures

